# High-Strength Organic–Inorganic Composites with Superior Thermal Insulation and Acoustic Attenuation

**DOI:** 10.1021/acspolymersau.3c00037

**Published:** 2024-01-16

**Authors:** Divya Iyer, Mohammad Galadari, Fernaldy Wirawan, Vanessa Huaco, Ricardo Martinez, Michael T. Gallagher, Laurent Pilon, Kanji Ono, Dante A. Simonetti, Gaurav N. Sant, Samanvaya Srivastava

**Affiliations:** † Department of Chemical and Biomolecular Engineering, University of California, Los Angeles, California 90095, United States; ‡ Department of Mechanical and Aerospace Engineering, University of California, Los Angeles, California 90095, United States; § Mattress Recycling Council, Alexandria, Virginia 22314, United States; ∥ Department of Bioengineering, University of California, Los Angeles, California 90095, United States; ⊥ Department of Materials Science and Engineering, University of California, Los Angeles, California 90095, United States; # Institute for Carbon Management, University of California, Los Angeles, California 90095, United States; ∇ Department of Civil and Environmental Engineering, University of California, Los Angeles, California 90095, United States; ○ California NanoSystems Institute, University of California, Los Angeles, California 90095, United States

**Keywords:** polyurethane upcycling, functional composites, high-strength composites, organic−inorganic hybrid materials, thermal insulation, acoustic attenuation

## Abstract

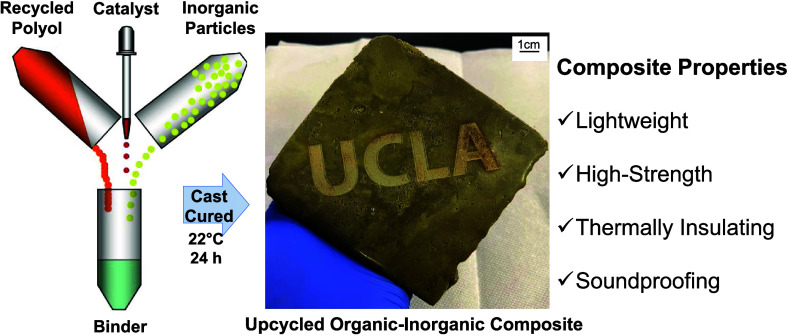

We demonstrate facile fabrication of highly filled, lightweight organic–inorganic composites comprising polyurethanes covalently linked with naturally occurring clinoptilolite microparticles. These polyurethane/clinoptilolite (PUC) composites are shown to mitigate particle aggregation usually observed in composites with high particle loadings and possess enhanced thermal insulation and acoustic attenuation compared with conventionally employed materials (e.g., drywall and gypsum). In addition to these functional properties, the PUC composites also possess flexural strengths and strain capacities comparable to and higher than ordinary Portland cement (OPC), respectively, while being ∼1.5× lighter than OPC. The porosity, density, and mechanical and functional properties of these composites are tuned by systematically varying their composition (diisocyanate, polyurethane, and inorganic contents) and the nature of the organic (reactivity and source of polyol) components. The fabrication process involves mild curing conditions and uses commonly available reagents (naturally occurring aluminosilicate particles, polyols, and diisocyanate), thereby making the process scalable. Finally, the composite properties are shown to be independent of the polyol source (virgin or recycled), underlining the generality of this approach for the scalable utilization of recycled polyols.

## Introduction

The creation of robust, highly filled composites comprising inorganic and organic phases remains a longstanding challenge. The primary inspiration for pursuing the fabrication of such hybrid materials comes from the exceptional properties of naturally occurring composites such as nacre (aragonite platelets bound together by proteins and mineral bridges)^1,2^ and clamshells (predominantly comprising calcium carbonate bound by chitin).^3^ The well-known toughness of these materials is attributed to their unique microstructure comprising lamellar arrangements of platelets, which prevents crack propagation under stress.^3−5^

Such natural composites have inspired research efforts to create synthetic organic–inorganic composites comprising inorganic materials as the majority phase to achieve enhanced physical, mechanical, or functional properties.^6−8^ However, the fabrication and properties of these nacre-like composites are sensitive to the nature of lamellar arrangements^6^ and often involve high sintering temperatures and rigorous processing conditions.^8^ Therefore, reproducing the lamellar arrangements of microparticles in a scalable manner is challenging in synthetic settings, stymied by the broad adoption of synthetic nacres.

In contrast, synthetic organic–inorganic composites with polymers as the primary matrix have become ubiquitous due to their ease of processing and favorable enhancements in mechanical (e.g., flexural, compressive, and tensile strengths) or functional (e.g., electrical or thermal conductivity, antibacterial properties, and flame resistance) properties.^9−11^ In such composites, the polymer (e.g., polypropylene, polyesters, and polyurethane) impart processability, and the inorganic components (e.g., minerals, aluminosilicates, graphite, and graphene) provide property enhancements.^9−11^ In recent years, polyurethane (PU) has emerged as a versatile primary matrix. PU is formed by an addition polymerization between a polyol and a diisocyanate; by varying the composition and reaction conditions, PU can be processed into dispersions, foams, and thermoplastic elastomers.^12^ Composites of PU with inorganic materials such as zinc oxide (and silver) (4–24 vol %),^13^ graphite (up to 20 wt %),^14,15^ and silver (10 wt %)^16^ have resulted in films, flexible and rigid foams with improved electrical conductivity and electromagnetic shielding ability, combustion resistance, and antibacterial properties.^13−16^ While these additives (up to 25–30 wt %) expand PU’s utility, further enhancements are limited due to particle aggregation and debonding-related deterioration in mechanical and functional properties.^14,15^

Recently, we demonstrated a facile method to overcome particle aggregation to fabricate highly filled (up to 60 wt %) lightweight PU-clinoptilolite composites by covalently linking micron-sized inorganic clinoptilolite particles (aluminosilicates) to organic polyols by aliphatic diisocyanate linkers (isophorone diisocyanate; IPDI).^17^ Our approach was inspired by strategies where grafting polymers to inorganic surfaces (e.g., polymer-calcium silicate hydrate^18^ and polymer-grafted silica nanoparticles^19,20^) led to enhanced compatibility between the organic and inorganic components, and significant improvements in mechanical properties. When compared to OPC, our composites exhibit lower density (up to 3×), comparable flexural strengths, and higher strain capacities (up to 5× OPC).^17^

Despite the promising enhancements in the flexural properties, our previous demonstration of the PU-clinoptilolite (PUC) composites suffered from strength deterioration due to increased porosity at high IPDI contents. Owing to its aliphatic nature, IPDI reacts slowly with hydroxyl groups while also being susceptible to side reactions that lead to foaming. While, on the one hand, a higher IPDI concentration was preferred to offset the low reactivity and obtain better organic–inorganic binding, on the other hand, the increased porosity led to weaker composites. Additionally, the influence of polyol reactivity and polymer content on the nature and mechanical, thermal insulation, and acoustic barrier properties of the PUC composites remains unknown, impeding the development of a generalized compositing approach. In this study, we address these research questions by expanding the scope of our composite fabrication approach and present functional characterizations of our lightweight, high-strength PUC composites to facilitate their use as lightweight thermal insulators and acoustic barriers.

Here, we fabricated composites with controlled porosity by utilizing an aromatic diisocyanate linker (toluene diisocyanate; TDI). Owing to its aromatic nature, TDI possesses a higher reactivity than the aliphatic IPDI,^12^ resulting in an early onset of curing and covalent network formation. This eliminated the need for elevated curing temperatures and led to improvements in strength at lower isocyanate contents (and higher solid loadings) than IPDI.^17^ With these modifications, herein, we present lightweight, high-strength PUC composites with controlled porosity. While the hybrid nature and covalent linkages contribute to the low density and high strength of these composites, their inherent porosity has been shown to lead to enhanced thermal insulation and acoustic attenuation properties compared to conventionally employed materials (e.g., OPC, drywall, and gypsum) that render them suitable for functional applications. Moreover, the physical, mechanical, and functional properties of composites comprising recycled polyols are shown to be similar to those of composites composed of virgin polyols, thus presenting an attractive methodology for appropriate utilization and upcycling of glycolyzed PU products without compromising the composite properties.

## Results and Discussion

### Room-Temperature Fabrication of Lightweight PUC Composites

**Figure 1:**
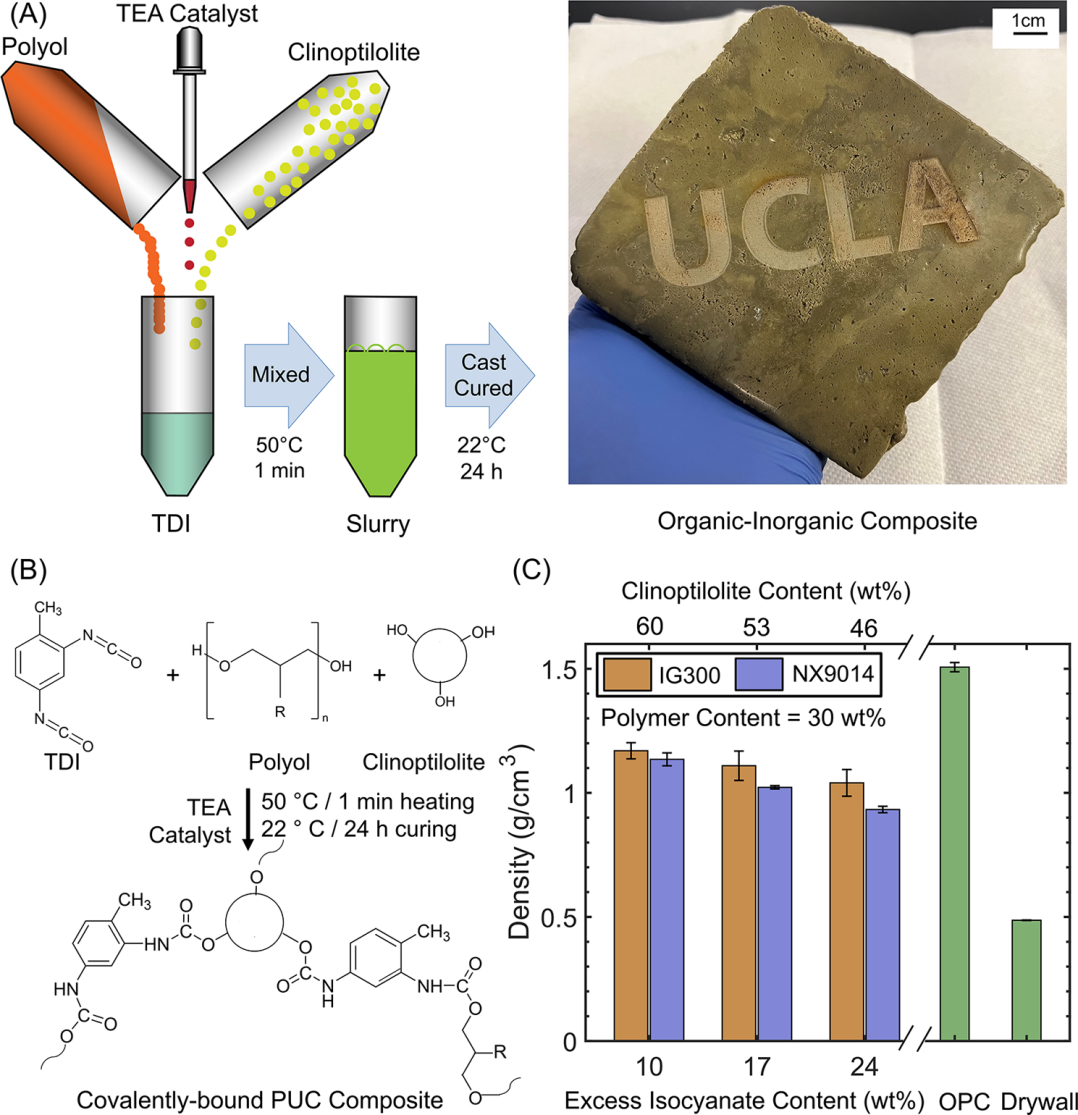
(A) Schematic representation of PUC composite fabrication involving the mixing of polyol (recycled or virgin), toluene diisocyanate linker (TDI), and inorganic clinoptilolite particles. The PUC composite shown here was laser-etched with the logo at the Lux Lab facility (UCLA Library). (B) Schematic portraying the covalent linkages between the organic (TDI, polyol) and inorganic (clinoptilolite) components. (C) Density of PUC composites, OPC, and gypsum drywall. The PUC composites comprise clinoptilolite, TEA, TDI, and IG300 polyol (orange) or NX9014 polyol (blue) at varying contents of excess isocyanate and clinoptilolite. The PU content was 30 wt % in all of the samples.

High-strength lightweight PUC composites were fabricated by covalently linking clinoptilolite (inorganic) particles and polyols (recycled or virgin) by using an aromatic organic binder (TDI). Information about the nature of the polyol and the PUC composite compositions is provided in Tables S1 and S2, respectively. The polyol was added to TDI, followed by clinoptilolite under constant mixing, prior to casting and curing at room temperature (Figure 1A). The reactive isocyanate groups on TDI initiate urethane linkages with OH groups from the polyol, as well as clinoptilolite particles, thus introducing compatibility between the organic (polyol) and inorganic (clinoptilolite) components and forming a PU network (Figure 1B).^21^ We note that while the high reactivity of TDI enhances the rate of covalent network formation, it is still susceptible to side reactions (with moisture or carboxylic acid groups), likely producing CO_2_ and leading to increased porosity in the cured composites.^17,22,23^ To aid the release of CO_2_ and minimize porosity, the mixture was heated at 50 °C for 1 min to accelerate the release of gas bubbles prior to curing at room temperature (Figure 1A). The higher reaction rate of TDI (compared with IPDI), coupled with the modified fabrication strategy, led to fewer entrapped gas bubbles and allowed room-temperature curing of the composites.

The density of the PUC composites (0.9–1.2 g/cm^3^) was markedly lower than OPC (1.5 g/cm^3^), albeit higher than drywall (0.5 g/cm^3^) (Figure 1C, Table S2). Decreasing the clinoptilolite content from 60 to 46 wt % (and concomitantly increasing the excess TDI content from 10 to 24 wt %, at a constant PU content of 30 wt %) led to decreasing density from 1.2 to 0.9 g/cm^3^ (Figure 1C).

The hybrid nature of these PUC composites (0.9–1.2 g/cm^3^) contributes to their low densities, compared to OPC (1.5 g/cm^3^). Despite the innately higher density of clinoptilolite (2–2.2 g/cm^3^),^24,25^ the low density of the PUC composites can be attributed to their porous structure and the presence of the organic components. The effect of porosity on the density is more pronounced at higher excess TDI contents (and lower clinoptilolite contents) owing to a lower solid loading and increased foaming. Lastly, similar densities of upcycled (IG300; OH-value = 290 mg/g) and virgin (NX9014; OH-value = 260 mg/g) composites arise from the comparable OH-value polyols, resulting in comparable microstructures emerging from their similar reactivity with NCO groups.

### Polyol OH-Value Dictates the Flexural Properties of PUC Composites

**Figure 2:**
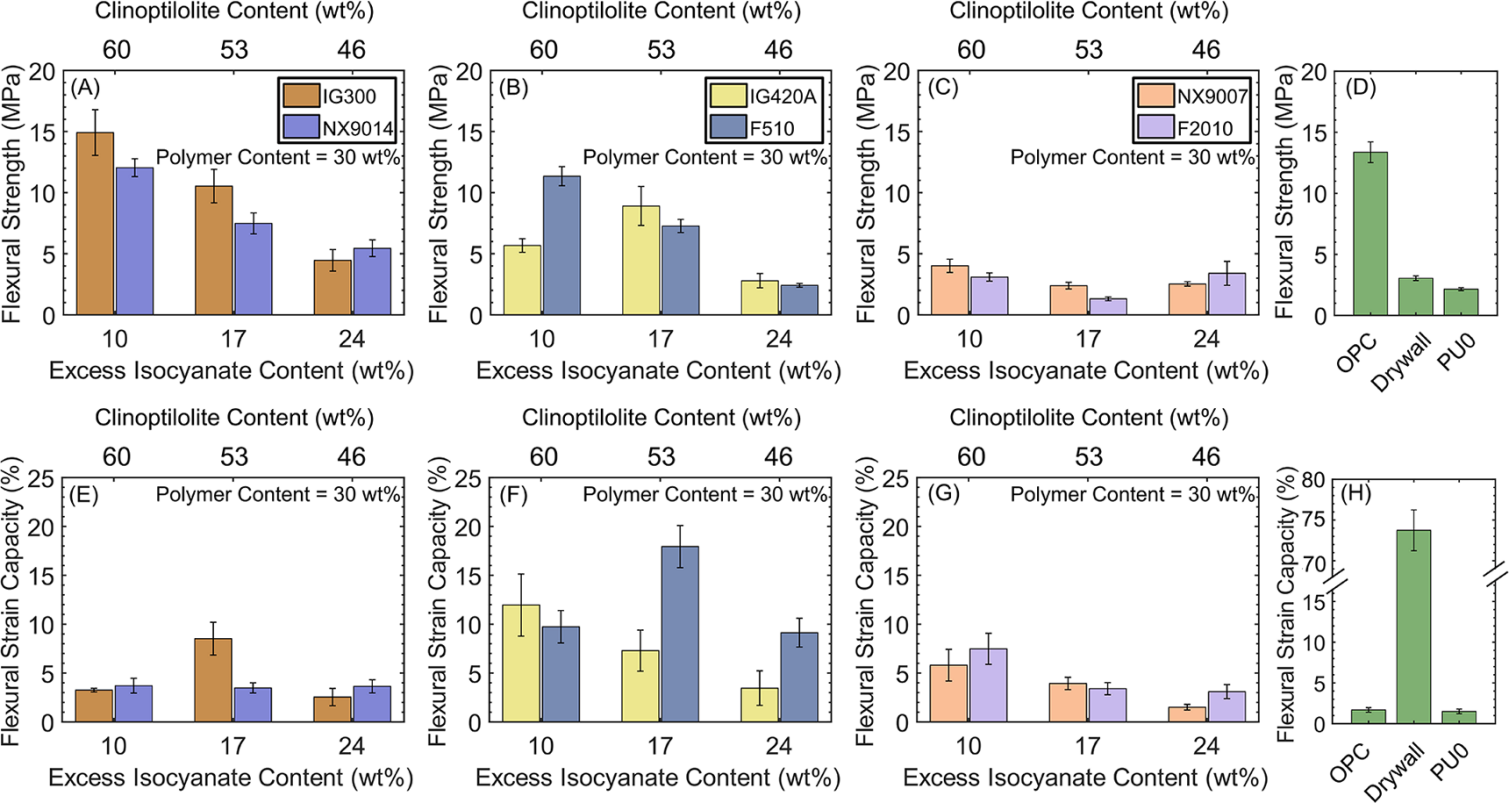
Flexural strengths and strain capacities of (A, E) medium-OH-value upcycled (IG300; orange) and virgin (NX9014; purple), (B, F) high-OH-value upcycled (IG420A; yellow) and virgin (F510; blue), and (C, G) low-OH-value virgin (NX9007; orange and F2010; purple) PUC composites with varying contents of clinoptilolite (60–46 wt %) and excess isocyanate (10–24 wt %). The polymer content was fixed at 30 wt %. (D, H) Flexural strengths and strain capacities of the OPC, drywall, and PU0 composites (no polyol; clinoptilolite particles bound with TDI).

The low density of the covalently linked PUC composites and their high inorganic loadings (46–60 wt %) present avenues for their application as structural materials. Figure 2 shows the flexural strengths (Figure 2A–D) and strain capacities (Figure 2E–H) of the PUC composites. The flexural strengths of PUC composites comprising medium-OH-value polyols (Figure 2A) compared well with OPC and markedly exceeded the strengths of drywall and PU0 composites (TDI-clinoptilolite; no polyol) (Figure 2D). The IG300 composite comprising 60 wt % clinoptilolite, 10 wt % excess isocyanate, and 30 wt % PU exhibited the highest flexural strength (15 MPa; 1.12× OPC); the strength of the composites consistently decreased with increasing excess TDI content to 17 and 24 wt % (and decreasing clinoptilolite content to 53 and 46 wt %, respectively, with 30 wt % PU content).

The OH-value of the polyols had a profound effect on the flexural performance of the composites. The flexural strengths of high-OH-value composites (Figure 2B) and low-OH-value composites (Figure 2C) were both lower than those of the medium-OH-value composites. While the high-OH-value composites followed similar flexural strength trends with composition as the medium-OH-value composites, the flexural strengths of low-OH-value composites were the lowest among all of the samples and were largely independent of composition.

The flexural strain capacities of medium-OH-value composites (3–9%, Figure 2E) were comparable to the low-OH-value composites (3–7%, Figure 2G) but were markedly lower than the high-OH-value composites (5–20%, Figure 2F). Across the three sets of OH-value composites, no specific trends with composition were identified. Notably, the strain capacities of all PUC composites were significantly higher than OPC (up to 5×) and the PU0 composite (up to 5.6×) but were lower than that of drywall (∼74% flexural strain capacity) (Figure 2H). Overall, the flexural strain capacities of the TDI-based composites consisting of medium-OH-value (virgin NX9014 and upcycled IG300) (Figure 2E) and low-OH-value (virgin NX9007 and F2010) (Figure 2G) composites were comparable to IPDI-based upcycled IG300 composites (4–11%).^17^

The extent of covalent linkage between the hydroxyl groups of the polyols and the clinoptilolite particle surfaces, facilitated by the reactive isocyanate groups from TDI, dictates the material attributes of the PUC composites (Figures 1C and 2). The comparable hydroxyl values (260 and 290 mg/g, respectively) and reactivity of virgin NX9014 and upcycled IG300, despite differences in their chemical backbone (Table S1), lead to composites with comparable flexural strengths (Figure 2A) and strain capacities (Figure 2E). Similar trends were noted for virgin F510 and upcycled IG420A composites (Figure 2B,F). We ascribe this similarity to a comparable extent of organic–inorganic network formation. More importantly, this similarity in material attributes enables a complete replacement of virgin polyols with recycled polyols without compromising the flexural properties of these hybrid composites.

The nonintuitive trends in flexural strengths and strain capacities with varying polyol OH-values require further scrutiny. While higher flexural strength of the medium-OH-value PUC composites (Figure 2A) compared to low-OH-value PUC composites (Figure 2C) was expected due to the higher reactivity of the former polyols, the lower flexural strengths of the high-OH-value PUC composites (Figure 2B) is unexpected. We posit that the formation of the PU network outdoes the development of the clinoptilolite–TDI linkages, causing a decline in flexural strength (Figure 2B) and an increase in flexural strain capacities (emerging from the robust PU network, Figure 2F) as compared to medium-OH-value (Figure 2E) and low-OH-value (Figure 2G) polyols. At the same time, the low strengths (Figure 2C) and strain capacities (Figure 2G) of the low-OH-value polyol (F2010, NX9007) composites can be attributed to the absence of sufficient OH groups for the formation of a robust network. The poor flexural strength (Figure 2D) and strain capacity (Figure 2H) of the PU0 (no polyol) composites underscore the role of polyols as ductility-enhancing components. As a reference, the OPC exhibits high flexural strength (Figure 2D) but suffers from low flexural strain capacity (Figure 2H) owing to its exclusively inorganic nature.

Replacing the aromatic TDI with aliphatic IPDI in the IG300 composites led to a decrease in flexural strengths, with the optimal composition shifting to 17 wt % excess isocyanate, 53 wt % clinoptilolite, and 30 wt % PU content.^17^ Using TDI also enabled room temperature curing compared to IPDI composites that required curing at 50 °C.^17^ The lower curing temperature and high strength of TDI-based PUC composites at a lower excess isocyanate content (10 wt %; Figure 2A) than the IPDI-based PUC composites can be attributed to the aromatic nature and resonance structures of TDI. These aspects contribute to its high reactivity and benefit the formation of covalent isocyanate linkages in the TDI-based composites to a greater extent than the aliphatic IPDI-based composites. The aromatic ring in TDI aids the delocalization of the negative charge and lowers the electron cloud density on the carbon atom, making it susceptible to nucleophilic attack (e.g., reaction with OH).^12^ However, the presence of an alkyl group (electron-donating) group on the aromatic ring can lower the reactivity of the second diisocyanate group.^12^ This effect is more pronounced in TDI than in IPDI; while both isocyanate groups are attached to the same aromatic ring in TDI, in the case of IPDI, they are separated by cycloaliphatic and aliphatic moieties.

The overall higher reactivity of TDI (as compared to aliphatic IPDI) with OH groups led to early initiation of curing, superseding the side reactions to a greater extent (likely due to the lower reactivity of the second isocyanate group in TDI). Owing to this, the flexural strengths of TDI composites are higher than similar IPDI composites while needing a lower excess isocyanate content and curing temperature.^23^ At higher excess isocyanate contents (and lower clinoptilolite contents), the extent of side reactions increases due to the lower availability of OH groups for organic–inorganic binding. This is confirmed by the increase in porosity (decrease in density; Figure 1C) and concomitant decrease in flexural strengths (Figure 2A–C) with increasing isocyanate contents.

### PUC Composites Exhibit MPa-Scale Compressive Strength

**Figure 3:**
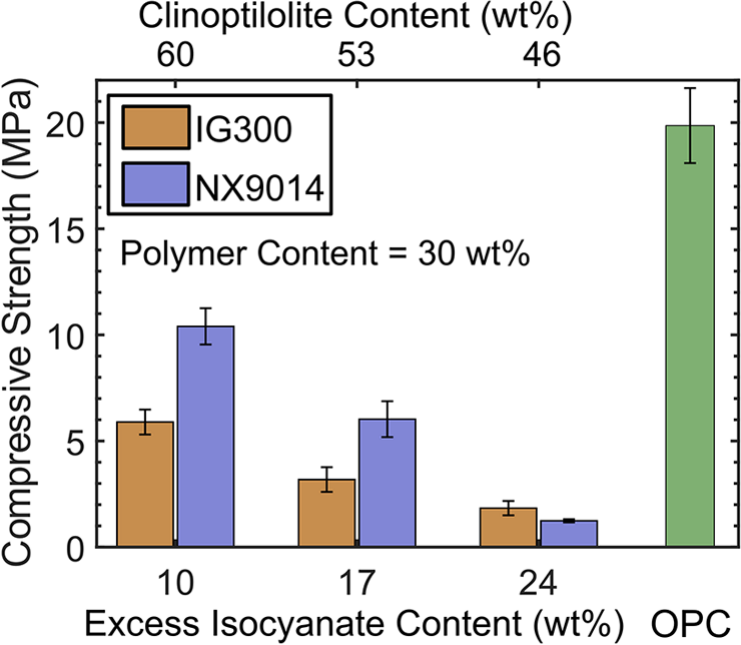
Compressive strength of recycled (IG300) and virgin (NX9014) PUC composites with varying excess isocyanate and clinoptilolite contents. Compressive strength of the OPC is also shown as a reference.

An important feature of structural materials is their ability to withstand uniform loads (compressive properties) in addition to bending stresses (flexural properties). Compressive properties are typically evaluated for materials used as support beams. Given the high flexural strengths (superior or comparable to those of OPC; Figure 2A,D) of the medium-OH-value PUC composites, we compared their compressive strengths with those of OPC (Figure 3) to benchmark their performance. The compressive strengths of upcycled (IG300) and virgin (NX9014) PUC composites were noted to be similar and were highest (0.5× OPC) for the composites with 10 wt % excess isocyanate content (clinoptilolite 60 wt %, PU 30 wt %). Similar to the flexural strength trends (Figure 2A), the compressive strength of the composites decreased with increasing excess isocyanate (and decreasing clinoptilolite) content, again attributable to a greater extent of side reactions leading to an overall decrease in organic–inorganic binding in addition to a lower inorganic loading. The comparisons between the flexural (Figure 2A) and compressive strengths (Figure 3) of the composites also reveal an interesting feature: the presence of the organic components (PU and isocyanate) causes a decrease in compressive strength of the composites owing to their lower load-bearing capacities but, at the same time, improves the flexural properties owing to their better stress dissipation characteristics. Lastly, the similar compressive strengths of virgin and upcycled composites again underscore the complete replaceability of virgin polyols with recycled polyols.

### Controlling Composite Microstructure with Excess Isocyanate Content

**Figure 4:**
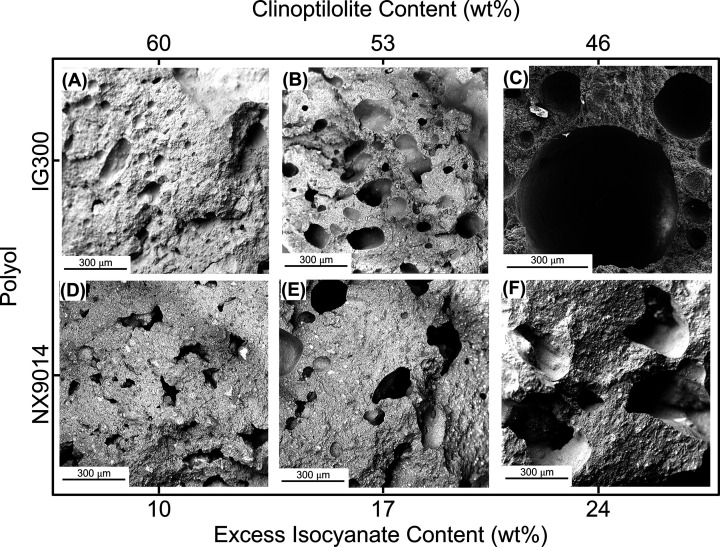
Scanning electron micrographs (SEM) of PUCs with (A, D) 10 wt %, (B, E) 17 wt %, and (C, F) 24 wt % excess isocyanate contents, comprising upcycled polyol (IG300, A–C) or virgin polyol (NX9014, D–F). A constant PU content of 30 wt % was maintained, while the clinoptilolite content was varied between 60 and 46 wt %.

The densities (Figure 1C, Table S2), flexural properties (Figure 2), and compressive strengths (Figure 3) of the PUC composites are dictated by the extent of organic–inorganic bonding and the side reactions. As noted earlier, the side reactions result in the evolution of CO_2_ during the curing process, resulting in voids and porosity in the composites and influencing their morphology. The morphology of medium-OH-value upcycled composites (IG300; Figures 4A–C and S1–S3) and virgin polyol composites (NX9014; Figures 4D–F and S4–S6), imaged using scanning electron microscopy at magnifications ∼250×, remained similar to increasing excess isocyanate content (10–24 wt %). The inorganic particles appeared to be uniformly distributed, indicating adequate binding between the inorganic and organic phases by TDI. This is a visible improvement to our prior work wherein agglomerates were noted at low (10 wt %) IPDI content.^17^

As is evident from the micrographs, the size and distribution of voids leading to porosity in the composites increased with increasing excess isocyanate contents (and decreasing clinoptilolite contents), emerging from the greater evolution of CO_2_ during the curing process. The low (and uniform) void distribution of the composites with 10 wt % excess isocyanate contents (Figure 4A,D) denotes adequate binding and is posited to contribute to these composites exhibiting slightly higher densities (1.13–1.17 g/cm^3^) and the highest flexural (Figure 2A) and compressive strengths (Figure 3). Simultaneously, the high porosity in composites with 17–24 wt % excess isocyanate contents (Figure 4C,F) led to their low density (0.93–1.05 g/cm^3^, Figure 1C, Table S2), as well as low flexural (Figure 2A) and compressive strengths (Figure 3). These observations confirm that the porosity (and consequently the physical and mechanical properties) of these PUC composites can be controlled by altering the composition. We harness this feature of PUC composites to expand their applicability, as discussed in a later section.

### Optimizing Polymer Content to Maximize Flexural Performance

After establishing the significant role of the inorganic component (clinoptilolite) and organic binder (excess isocyanate content) in determining the physical and mechanical properties of the PUC composites, we turned our attention to the role of the organic PU network in enhancing ductility and fracture resistance of the composites. The absence of a polymer network will result in brittle composites, as confirmed by the low flexural strength (Figure 2D) and strain capacity (Figure 2H) of PU0 (clinoptilolite particles bound by TDI; no polyol).

**Figure 5:**
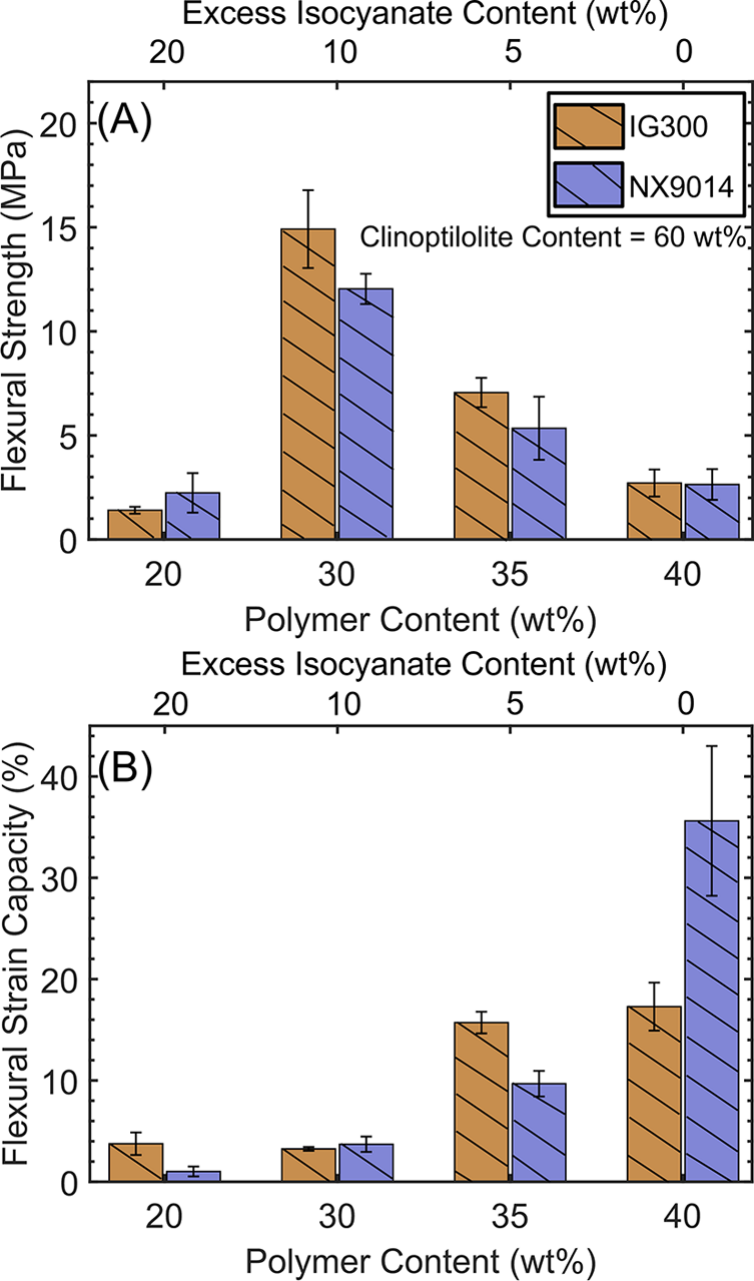
(A) Flexural strength and (B) flexural strain capacities of the upcycled (IG300) and virgin (NX9014) PUC composites with varying PU content (20–40 wt %) at fixed clinoptilolite loading (60 wt %).

The role of the PU network was isolated by systematically varying the PU content (with corresponding adjustments in the excess TDI content) in the most inorganic-dense (60 wt % clinoptilolite content) medium-OH-value PUC composites (virgin NX9014 and recycled IG300). With increasing PU content (from 20 to 30 wt %), the flexural strengths of the PUC composites increased by almost an order of magnitude, followed by a steady decrease at higher PU contents (35 and 40 wt %) (Figure 5A). Interestingly, the composite with 20 wt % PU contained the maximum excess TDI content (20 wt %; Table S3), yet it possessed the lowest flexural strengths (Figure 5A). Additionally, despite having no excess TDI content, the flexural strengths of composite with 40 wt % PU were higher than those with 20 wt % PU. Notably, the flexural strengths of composites with PU contents away from the optimal composition (30 wt % PU content) were significantly lower than those of OPC (Figure 2D).

The flexural strain capacities of the PUC composites did not exhibit any significant variation upon increasing the PU content in the composites from 20 to 30 wt % but increased progressively upon increasing the PU content further up to 40 wt %. These high flexural strain capacities in the composites with 35 or 40 wt % PU contents (Figure 5B) were accompanied by a decrease in flexural strengths (Figure 5A), yielding weak but flexible composites.

These variations in the flexural strengths and strain capacities of the composites with varying PU contents emphasize the role of the PU network in determining the composite properties. The lowest flexural strengths and strain capacities of the composites with 20 wt % PU can be ascribed to the greater extent of foaming (and porosity) due to high excess isocyanate content (20 wt %) (Figure 5A). Contrary to this, with increasing polymer contents (35 and 40 wt %) and decreasing availability of excess isocyanate (5 and 0 wt %, respectively), a consistent decrease in strength (Figure 5A) was observed owing to insufficient binding between the organic and inorganic components. Composites with 30 wt % PU comprised an optimal balance between porosity (due to side reactions) and sufficient organic–inorganic linkages, resulting in the highest flexural strengths. At the same time, the increase in flexural strain capacities at higher PU contents reflects the influence of the organic network in determining the flexibility of the composites (Figure 5B). As the polymer content increased (and the excess isocyanate content decreased), the formation of PU linkages took precedence over organic–inorganic linkages, resulting in higher flexural strain capacities (Figure 5B) but lower flexural strengths (Figure 5A).

### Thermal Insulation and Sound Attenuation Are Dependent on the Composite Composition and Porosity

In addition to being promising candidates for structural applications owing to their robust flexural and compressive properties, the hybrid nature of these covalently linked organic–inorganic composites and their controlled porosity also render them suitable as thermal insulators and sound attenuators.

**Figure 6:**
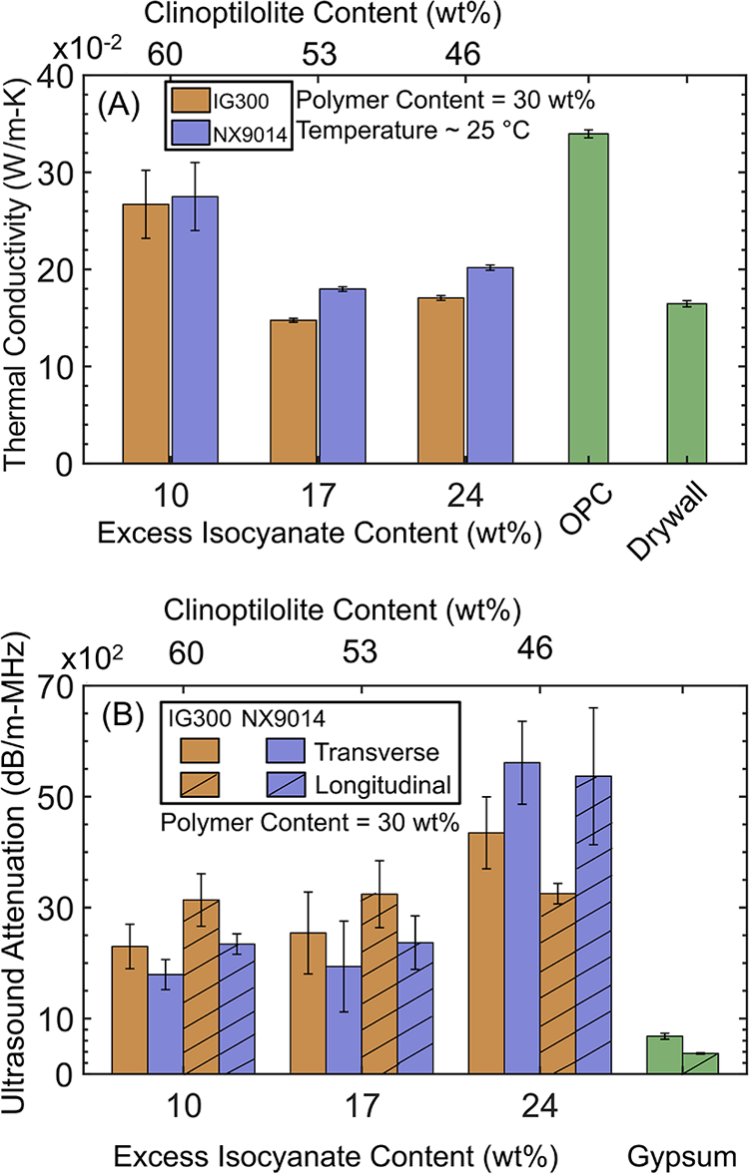
(A) Thermal conductivity and (B) ultrasound attenuation performance of upcycled (IG300) and virgin (NX9014) PUC composites have been compared to those of OPC and drywall (thermal conductivity) and gypsum (sound attenuation).

Overall, the thermal conductivity of the medium-OH-value PUC composites across compositions consistently remained lower than OPC and comparable to drywall (Figure 6A). Composites with 10 wt % excess isocyanate exhibited the highest thermal conductivity (poorest thermal insulation); the conductivities of composites with 17 and 24 wt % excess isocyanate were about 50% lower, while being comparable to each other and to drywall (Figure 6A). The low thermal conductivity of PUC composites with 17 and 24 wt % excess isocyanate contents can be ascribed to their increased porosity (Figure 4) and corresponding insulation from the trapped air, as well as lower inorganic contents.

The hybrid organic–inorganic PUC composites also performed as excellent acoustic barriers compared to gypsum, which is the primary component of drywall used for soundproofing (Figure 6B). The transverse and longitudinal ultrasound attenuation behaviors of the upcycled and virgin PUC composites were overall significantly higher than that of gypsum (Figure 6B). The transverse and longitudinal ultrasound wave attenuation values were both determined since ultrasound waves traverse in solid media along both these directions.^26^ Composites with 24 wt % excess isocyanate had the highest ultrasound attenuation (Figure 6B), with transverse and longitudinal attenuation being >6.4× and >8.8× than gypsum, respectively.

The increase in ultrasound attenuation with increasing excess isocyanate content (Figure 6B) corroborated the increased porosity of the PUC composites (Figure 4). The transmitted ultrasound wave energy is likely dissipated due to absorption by the composite material, in addition to scattering inside the pore walls.^27^ Therefore, a greater degree of porosity will correspond to enhanced ultrasound attenuation, concomitant with our observations for the PUC composites (Figure 6B).^28,29^

## Conclusions: Putting the Properties of PUC Composites into Perspective

Advancements in materials science have led to a significant expansion of the functionality of composites catering to a multitude of applications.^1,17,30−64^ Here, we have demonstrated the fabrication of lightweight, high-strength PUC composites comprising naturally occurring aluminosilicates, recycled (or virgin) polyol, and organic binder. The physical, mechanical, and functional properties of these highly filled composites (46–60 wt % inorganic particle loading) are shown to be controlled by varying the chemical composition, as well as the nature of organic and inorganic components. By optimizing these factors, composites with flexural strengths (up to 1.12× OPC) and strain capacities (up to 10.6× OPC) superior to OPC, as well as superior thermal insulation (2× OPC, 1.2× drywall) and acoustic attenuation (up to ∼15× gypsum), are fabricated.

**Figure 7:**
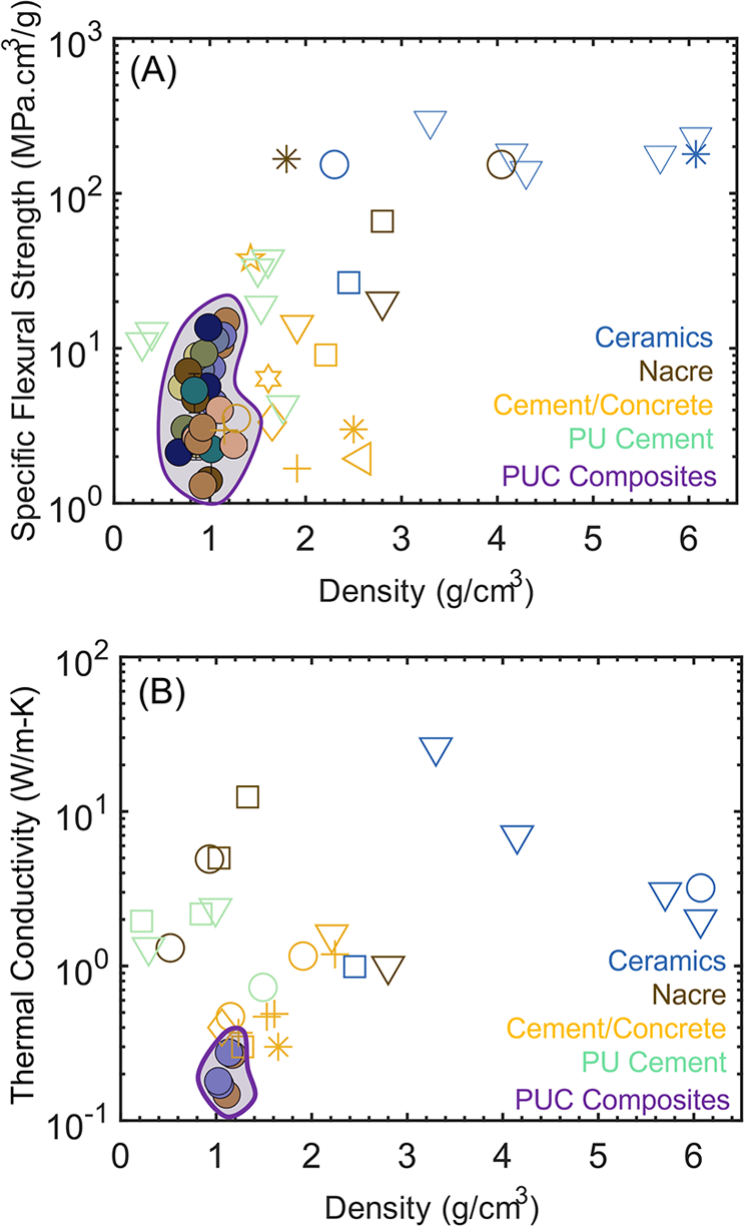
Evolution of (A) specific flexural strengths and (B) thermal conductivities of PUC composites with density, juxtaposed with other classes of materials discussed in the literature (Tables S4 and S5).

Encouraged by these findings, we juxtapose the specific flexural strengths (Figure 7A) and thermal conductivity values (Figure 7B) of the PUC composites against composites discussed in the literature (relevant citations have been listed in Tables S4 and S5).^1,17,30−57,59−64^ The specific flexural strengths of our PUC composites are comparable (or superior) to those of cement (or concrete)-based and PU-cement composites (Figure 7A) while also being lighter (Figure 7A). It must be noted that cement-based systems rely on hydration lasting over days (or weeks) to achieve high strengths. In contrast, our PUC composites are cured for 24 h and yield comparable strengths. Additionally, while prior contributions^51,59−61,65^ have reported the fabrication of PU-cement (or concrete) composites, the strengths arise primarily from the calcium silicate hydrate (C–S–H), complemented by the PU network. Our fabrication strategy utilizes covalent linkages between naturally occurring aluminosilicates and recycled (or virgin) polyols.

While ceramics^31−33,57^ and nacre-inspired^1,30,62−64^ composites are known to possess high flexural strengths, they are also significantly higher in density than our lightweight PUC composites (Figure 7A). The high strength of ceramics arises from their ordered structure and the presence of strong ionic and covalent bonds. Similarly, composites mimicking nacre harness the lamellar arrangement to attain high strengths (Figure 7A).^1,30,62−64^ However, the predominantly inorganic nature of both of these classes of materials contributes to their high densities. Interestingly, despite high solid loadings, our PUC composites are significantly lower in density due to their porous and hybrid nature.

In addition to favoring low densities, the controlled porosity in these PUC composites imparts high thermal insulation (low thermal conductivity; Figure 6A) and ultrasound attenuation (Figure 6B) abilities. Notably, in comparison to the other classes of materials shown in Figure 7B, the PUC composites have the lowest thermal conductivity values owing to their hybrid organic–inorganic nature and controlled porosity. Overall, the PUC composites fabricated in this work can be tuned to perform at strengths (Figures 2A–C and 7A) comparable to those of lightweight structural materials (Figure 1) while also demonstrating excellent thermal insulation (Figures 6A and 7B) and sound attenuation (Figure 6B) performance. Importantly, our methodology can be employed for raw materials sourced from varied (e.g., virgin vs depolymerized polyols) sources to obtain composites with comparable set of properties, thereby providing a multitude of opportunities for the appropriate utilization and sequestration of recycled polymer products.

## Experimental Design/Materials and Methods

### Materials

The polyols used in this study included virgin (NX9014, NX9007, F510, and F2010) and recycled (IG300 and IG420A) polyols (Table S1). The polyols have been categorized, based on their OH-values, as low (85–175 mg/g), medium (260–290 mg/g), and high (332–385 mg/g). Additionally, we note that recycled polyols (Emery Oleochemicals, LLC) IG300 (OH-value 290 mg/g) and IG420A (OH-value 385 mg/g) had OH-values comparable to virgin polyols NX9014 (OH-value 260 mg/g) and F510 (OH-value 332 mg/g), respectively. NX9007 (OH-value 175 mg/g) and NX9014 were supplied by the Cardolite Corporation. F510 (OH-value = 332 mg/g) and F2010 (OH-value = 85 mg/g) were supplied by Kuraray Co., Ltd. The specifications of these polyols are given in Table S1. Toluene diisocyanate (TDI) was obtained from Fisher Scientific. Triethylamine (TEA) was obtained from Millipore Sigma. Clinoptilolite (<2 μm particles, manufacturere specifications) was obtained from Heiltropfen Lab. Gypsum-based drywall was obtained from Stella Sealants.

### Composite Preparation

The preparation procedure for the PUC composites was similar to our previous study,^17^ with relevant modifications.^66^ Preweighed amounts of polyol and clinoptilolite particles were mixed with a measured volume of TDI in a centrifuge tube. Adequate mixing and particle surface wetting was facilitated by vortex mixing the mixure at regular intervals, followed by the addition of the TEA catalyst and further mixing to produce composite precursor pastes. Foaming was typically observed in the mixture; the release of gas bubbles was facilitated by heating the uncapped tube at 50 °C for 1 min. As previously discussed,^17^ drying the particles under vacuum or the polyol over a catalyst bed did not ameliorate foaming. The compositions of the PUC composites are listed in Table S2. The clinoptilolite content refers to the inorganic loading, while the polymer content is the organic content formed by the reaction of TDI with polyol (NCO/OH = 1:1 mol ratio). The excess TDI (or isocyanate) content refers to the TDI content available for a reaction with the clinoptilolite particles.

Composite samples for flexural tests were prepared by curing (24 h) the paste in a silicone mold of dimensions 70 mm × 10 mm × 6 mm (length × width × thickness) at room temperature and subsequently storing the cured samples in airtight containers. OPC samples of the same dimensions were prepared using type III OPC and mixing with water in a water-to-cement ratio of 0.4 (w/w), followed by curing for 24 h. Cubic samples (3 or 5 cm in length) for compressive strength tests were prepared by employing a mechanical stirrer to mix the precursor pastes and curing them in appropriate molds. Compressive strength tests were performed on 3 cm (each side) cubes for the NX9014 samples with excess isocyanate contents of 10 and 17 wt %, while all of the other measured cubic samples (composites, OPC) had a dimension of 5 cm (each side). The sample dimensions were accounted for in the calculations for compressive strength.

Composite (and OPC) samples for thermal conductivity measurements were prepared using a similar methodology, followed by curing (72 h) in a silicone mold with dimensions 152 mm (length) × 152 mm (width) × 25 mm (thickness). The sample surfaces were polished to be flat using an electric belt sander. Owing to rapid curing, high inorganic loading (60 wt %), and scale-up limitations at low excess isocyanate content (10 wt %), smaller disk-shaped samples (5–6 mm diameter, 50 mm thickness) were prepared for both polyols (NX9014, IG300) at that composition. Thermal conductivity measurements on the gypsum drywall sample were performed without any modifications to the received product. Sound absorption measurements were performed on samples cured (24–48 h) in a silicone mold (20 mm length × 20 mm width × 5 mm thickness). Prior to measurements, these samples were progressively fine-polished with sandpapers of varying grits (60–500).

### Density Measurements

The density of the cured composites was calculated by using the following equation
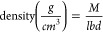
1where *M* is the mass of the composite sample (g) and *l*, *b*, and *d* refer to the sample length (cm), width (cm), and thickness (cm). Mean values of triplicate measurements have been reported; errorbars represent standard errors.

### Flexural Strength and Strain Capacity

Three-point bending (flexural) tests on the composite, OPC and drywall samples were performed using an Instron 5564. A span length of 20 mm and a strain rate of 0.5 mm/min were maintained. Using the load–displacement curve (Figure S7), the flexural strength and strain capacities were calculated using the following equations
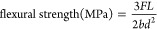
2
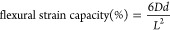
3where *F* refers to the maximum load prior to failure (N), *L*, *b*, and *d* refer to the span length (20 mm), sample width (mm) and the sample thickness (mm), respectively, while *D* refers to the displacement (mm) at *F*. Mean values of triplicate measurements have been reported; errorbars represent standard errors.

### Compressive Strength

Compressive strengths of the composites and the OPC were measured on a Test Mark Industries CM-3000 instrument equipped with a TS17 indicator. During measurements, the samples were placed in the center of the sample holder and compressed at 1 kN/s. Compressive strengths were calculated by using the equation
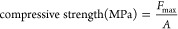
4where *F*_max_ and *A* refer to the maximum load prior to failure (N) and the exposed surface area (mm^2^), respectively. Mean values of triplicate measurements have been reported; errorbars represent standard errors.

### Microscopy

Fractured composites obtained from flexural tests were imaged by using either a Phenom XL G2 Desktop scanning electron microscope (SEM) or a ZEISS Supra 40VP SEM at the point of failure. The specimens were mounted on a stainless pin using a conductive carbon adhesive tape and observed under the SEM at an accelerating voltage of 10 kV and 250× magnification.

### Thermal Conductivity

Effective thermal conductivities were measured at room temperature using an in-house built apparatus conforming to ASTM C177-19 standards and operating on a single-sided mode (for smaller-sized samples with 10 wt % excess isocyanate).^67^ The setup comprised hot (final temperature *T*_h_) and cold (constant temperature *T*_c_) plates made of copper embedded with thermocouples; the sample was placed between these plates. The cold plate temperature was maintained constant using a recirculating chiller pumping a steady stream of cooling fluid, while the hot plate temperature *T*_h_ was achieved by employing a digital power supply to resistively heat its metered section (inner side) and guard section (outer side). Heat losses to the environment were minimized by insulating the setup with glass wool. The thermal conductivity was calculated as follows^67^
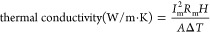
5where *I*_m_ and *R*_m_ refer to the current (A) supplied to and the resistance (ohm) of the metered section, while *A* is its area (m^2^). The term Δ*T* corresponds to the measured temperature difference between the hot (*T*_h_) and the cold (*T*_c_) plates. In practice, Δ*T* exceeded 10 °C and measurements were performed 3–4 times and averaged.

The thermal conductivity measurements for the large samples (152 mm × 152 mm × 25 mm) were obtained from the commercial instrument HFM 446 Lambda by Netzsch, also implementing the guarded hot plate method. Here, the temperature difference between the hot and cold plates was fixed at 10 °C and the mean temperature was varied between 5 and 90 °C. At each set point temperature, data was collected upon equilibration. The thermal conductivity was determined as follows
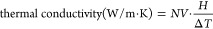
6where *N*, *V*, and *H* refer to the correction factor against borosilicate glass (standard reference), the average voltage supplied to the upper and lower plates, and the sample thickness, while Δ*T* refers to the temperature difference imposed between the hot and cold plates.

### Ultrasound Attenuation

Transverse and longitudinal ultrasound attenuation measurements on the PUC composites and gypsum were performed by employing a pair of 2.25 MHz Olympus V154RM ultrasonic transducers and a pair of 5 MHz Olympus V109RM ultrasonic transducers (13 mm contact diameter) for the transverse and longitudinal modes, respectively, in addition to a pulser and a digital oscilloscope (PicoScope 5242D).^68−70^ Alternate longitudinal attenuation measurements performed using other transducer pairs (1 MHz V103RM or 2.25 MHz V106RM) did not impact the results significantly. From the curves obtained for the attenuation coefficient (dB) vs frequency (MHz), the ultrasound attenuation values were determined (slope of the best-fit line). The attenuation for a sample of known thickness (in dB) was calculated using the following equation
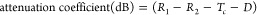
7where *R*_1_ (dB) and *R*_2_ (dB) refer to the face-to-face signal (no sample) and received signal (with sample), respectively, post a fast Fourier transform applied using Noesis software. *T*_c_ and *D* refer to the transmission coefficient and diffraction correction (expressed in the dB scale), respectively. These terms were obtained using the following equations^68−72^
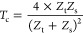
8
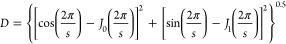
9where *Z*_t_ (MKS Rayl) and *Z*_s_ (MKS Rayl) refer to the acoustic impedance of the transducer and the acoustic impedance of the test sample, respectively.  and  are the Bessel functions of zeroth and first orders in *s*, with *s* being the Seki parameter obtained by the following equation^68−72^
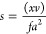
10where *x*, *v*, and *a* refer to the sample thickness (mm), wave velocity (longitudinal or transverse; mm/μs), and the transducer contact radius (mm), respectively, while *f* is the wave frequency (MHz). Transverse and longitudinal attenuation coefficients (dB/m-MHz) were obtained by normalizing the calculated attenuation values against the sample thickness (m).

## Supporting Information Available

Physical properties of polyols; composite compositions and densities; SEM micrographs of composites; reference values for properties of different classes of materials; and load–displacement curves for flexural strengths (PDF)
