# Supplementary material for: High-Strength Organic–Inorganic Composites with Superior Thermal Insulation and Acoustic Attenuation
Source: ACS Polym Au. 2024 Jan 16;4(1):86–97. doi: 10.1021/acspolymersau.3c00037 (PMC10870751; doi:10.1021/acspolymersau.3c00037)
Supplement: Supplementary file 1 — lg3c00037_si_001.pdf [file lg3c00037_si_001.pdf]

Supporting Information for

High Strength Organic-Inorganic Composites With Superior Thermal Insulation and Acoustic Attenuation

Divya Iyer,[1†] *Mohammad Galadari,* [1†] *Fernaldy Wirawan,* [1] *Vanessa Huaco,* [1] *Ricardo Martinez,*[2] *Michael T. Gallagher,*[3] *Laurent Pilon,*[2,4] *Kanji Ono,*[5] *Dante A. Simonetti,* [1,6] *Gaurav N. Sant,*[6,7,8] *Samanvaya Srivastava*[1,6,8*]

[1] Department of Chemical and Biomolecular Engineering, University of California, Los Angeles, CA 90095, USA
[2] Department of Mechanical and Aerospace Engineering, University of California, Los Angeles, CA 90095, USA
[3] Mattress Recycling Council, Alexandria, VA 22314, USA
[4] Department of Bioengineering, University of California, Los Angeles, CA 90095, USA
[5] Department of Materials Science and Engineering, University of California, Los Angeles, CA 90095, USA
[6] Institute for Carbon Management, University of California, Los Angeles, CA 90095, USA
[7] Department of Civil and Environmental Engineering, University of California, Los Angeles, CA 90095, USA
[8] California NanoSystems Institute, University of California, Los Angeles, CA 90095, USA
[†]Equal contribution
*Corresponding authors: S. Srivastava, Email: samsri@ucla.edu

Table of Contents:

Supplementary Tables S1-S5
Supplementary Figures S1-S7

OH-value category	Polyol	Type	OH value (mg KOH/g polyol)	Viscosity (cP)	Molecular weight (g/mol)	Source
Low	F2010	Polyester	85	7200	2000	Virgin
	NX9007	CNSL, branched, polyester-polyether	175	2900	1000	Virgin, biobased
Medium	NX9014	Non–CNSL, branched, polyester-polyether	260	1200	1000	Virgin, biobased
	IG300	Polyester-polyether	290	4000	-	Recycled
High	F510	Polyester	332	2200	500	Virgin
	IG420A	Polyester-polyether	385	1100	-	Recycled

Table S1: Polyol data sheet as provided by the manufacturers. CNSL refers to Cashew Nutshell Liquid.

Sample ID	Polyol	Clinoptilolite (wt%)	Polymer (wt%)	Excess TDI (wt%)	Density (g/cm^3)
F2010-I10	F2010 (85 mg/g)	60	30	10	0.92 ± 0.06
F2010-I17		53	30	17	0.93 ± 0.01
F2010-I24		46	30	24	0.76 ± 0.02
NX9007-I10	NX9007 (175 mg/g)	60	30	10	1.1 ± 0.06
NX9007-I17		53	30	17	1.25 ± 0.14
NX9007-I24		46	30	24	0.88 ± 0.04
NX9014-I10	NX9014 (260 mg/g)	60	30	10	1.13 ± 0.03
NX9014-I17		53	30	17	1.02 ± 0.01
NX9014-I24		46	30	24	0.93 ± 0.01
IG300-I10	IG300 (290 mg/g)	60	30	10	1.17 ± 0.03
IG300-I17		53	30	17	1.11 ± 0.06
IG300-I24		46	30	24	1.04 ± 0.05
F510-I10	F510 (332 mg/g)	60	30	10	1.06 ± 0.01
F510-I17		53	30	17	0.9 ± 0.03
F510-I24		46	30	24	0.87 ± 0.03
IG420A-I10	IG420A (385 mg/g)	60	30	10	0.71 ± 0.02
IG420A-I17		53	30	17	0.87 ± 0.01
IG420A-I24		46	30	24	0.86 ± 0.03

Table S2: Compositions of PUC composites comprising virgin (F2010, NX9007, NX9014, F510) and recycled polyols (IG300, IG420A) with varying OH-values.

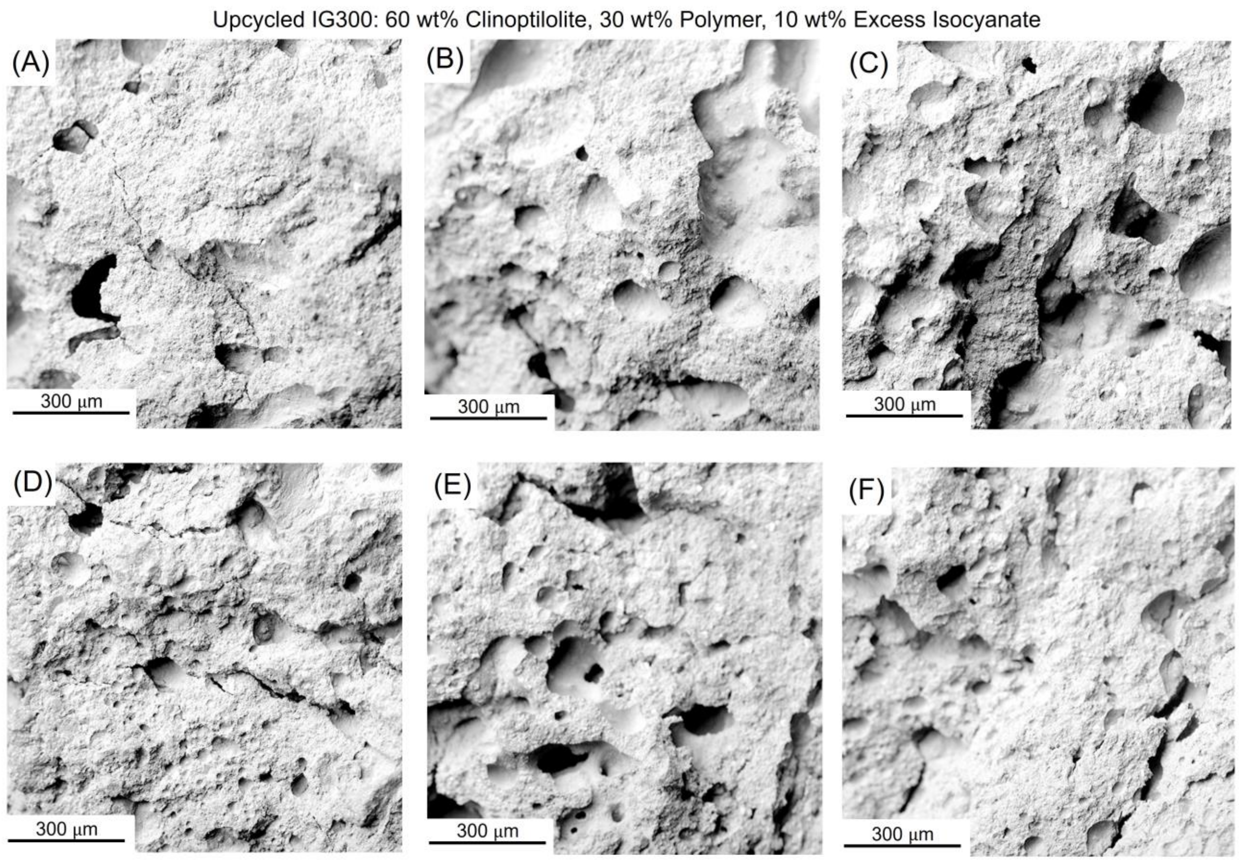

Figure S1: (A)-(F) SEM micrographs of upcycled (IG300) composite with clinoptilolite (60 wt%), polyurethane (30 wt%) and excess isocyanate (10 wt%).

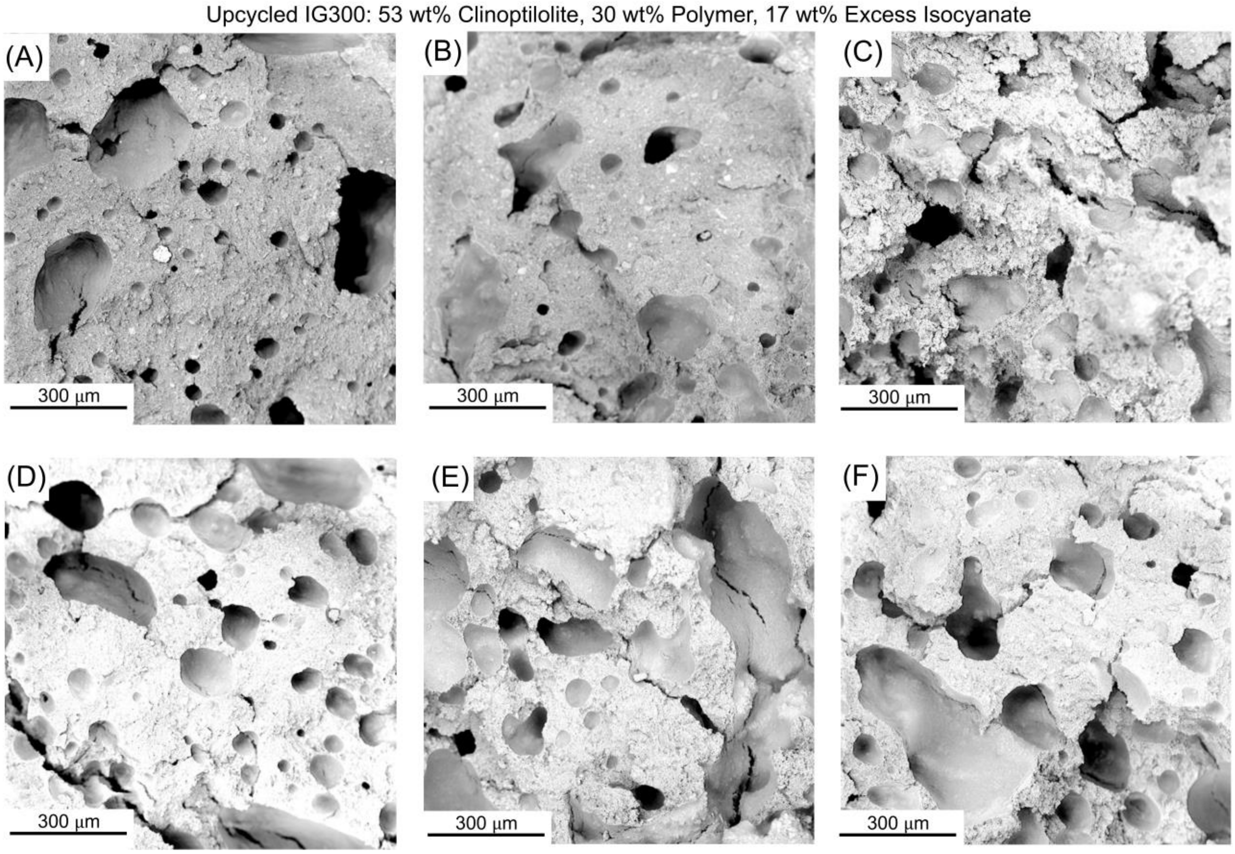

Figure S2: (A)-(F) SEM micrographs of upcycled (IG300) composite with clinoptilolite (53 wt%), polyurethane (30 wt%) and excess isocyanate (17 wt%).

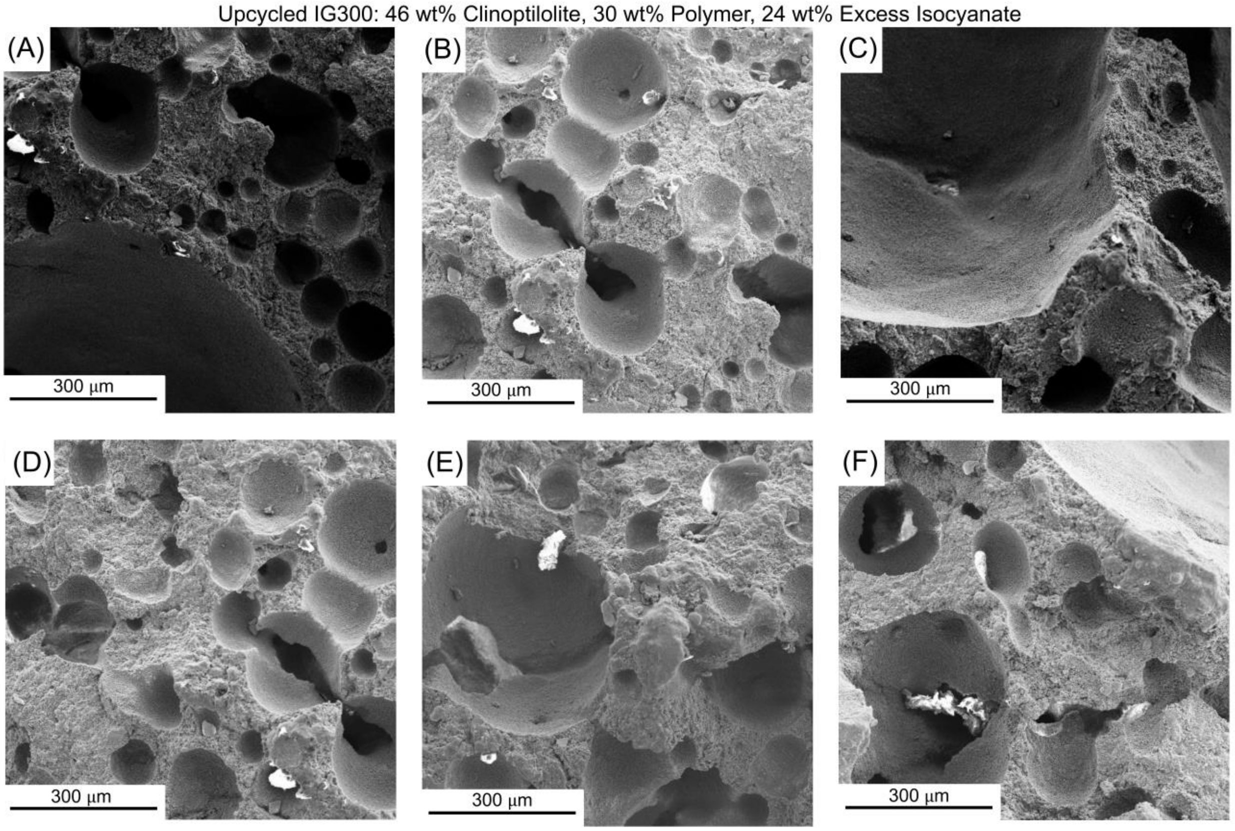

Figure S3: (A)-(F) SEM micrographs of upcycled (IG300) composite with clinoptilolite (46 wt%), polyurethane (30 wt%) and excess isocyanate (24 wt%).

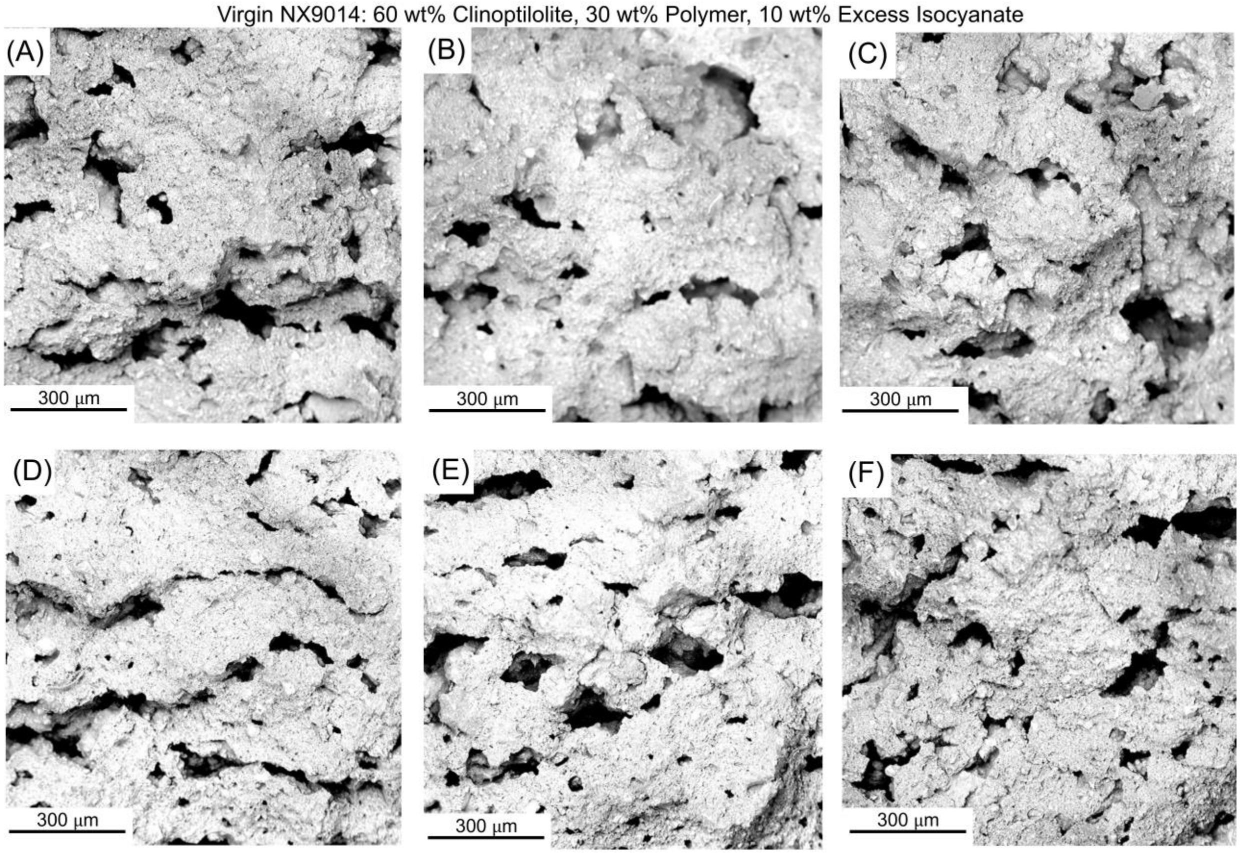

Figure S4: (A)-(F) SEM micrographs of virgin (NX9014) composite with clinoptilolite (60 wt%), polyurethane (30 wt%) and excess isocyanate (10 wt%).

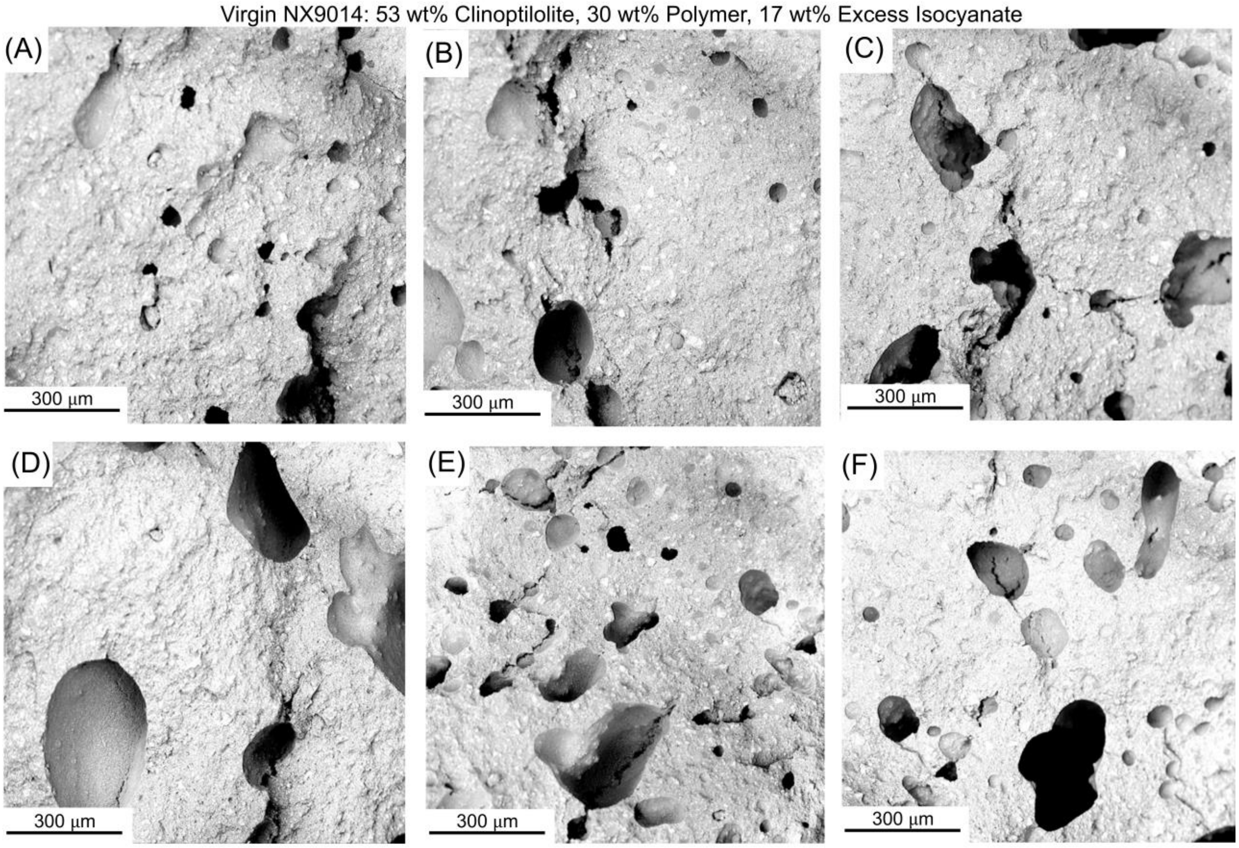

Figure S5: (A)-(F) SEM micrographs of virgin (NX9014) composite with clinoptilolite (53 wt%), polyurethane (30 wt%) and excess isocyanate (17 wt%).

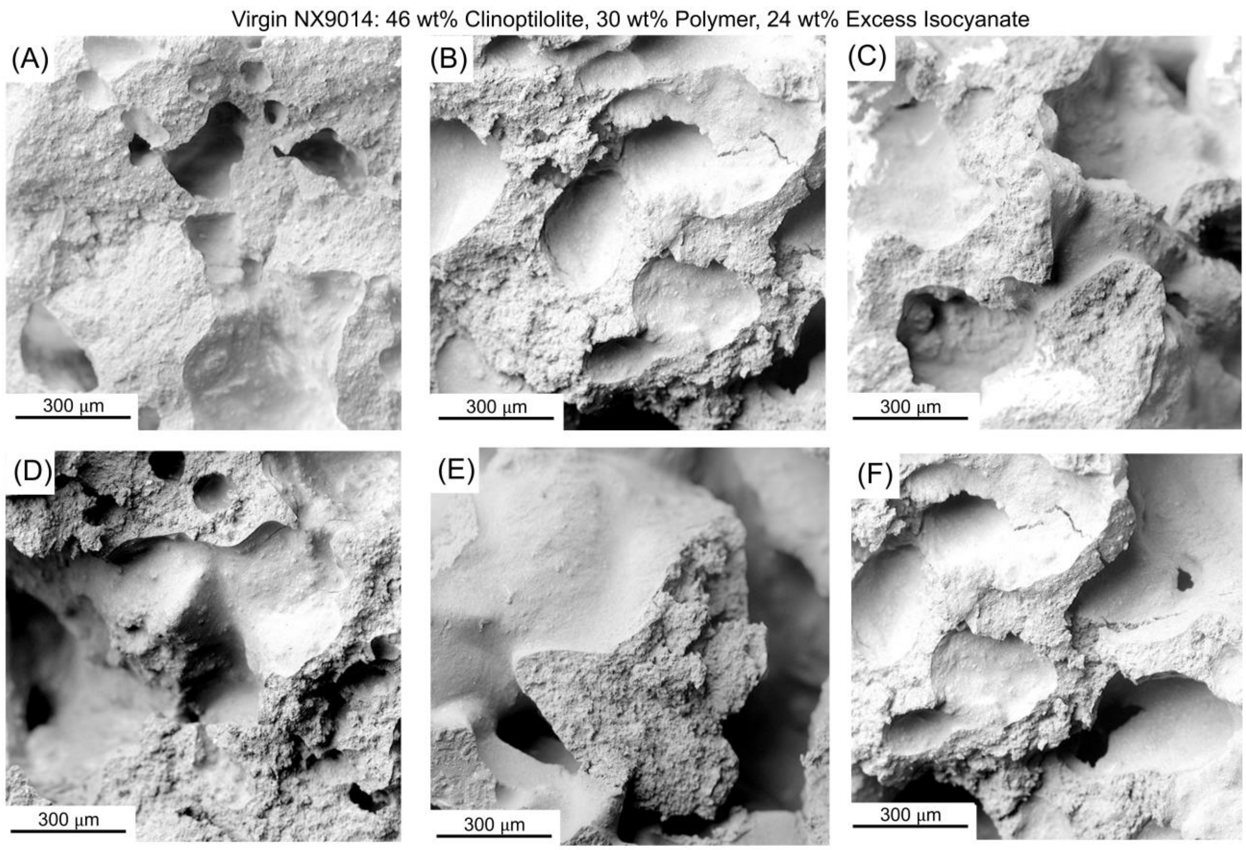

Figure S6: (A)-(F) SEM micrographs of virgin (NX9014) composite with clinoptilolite (46 wt%), polyurethane (30 wt%) and excess isocyanate (24 wt%).

Sample ID	Polyol	Clinoptilolite (wt%)	Polymer (wt%)	Excess TDI (wt%)
NX9014-P20	NX9014	60	20	20
NX9014-P30		60	30	10
NX9014-P35		60	35	5
NX9014-P40		60	40	0
IG300-P20	IG300	60	20	20
IG300-P30		60	30	10
IG300-P35		60	35	5
IG300-P40		60	40	0

Table S3: Compositions of PUC composites comprising virgin (NX9014) and recycled (IG300) polyols, at constant clinoptilolite content (60 wt%) and varying polymer (20–40 wt%) and excess isocyanate (0–20 wt%) contents.

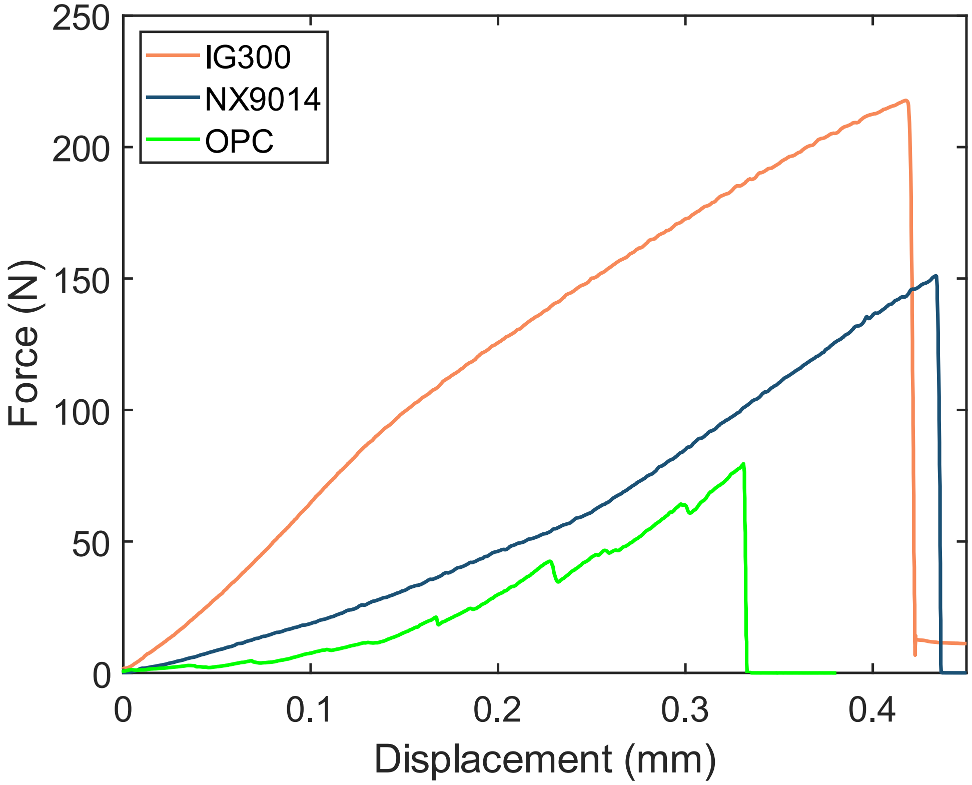

Figure S7: Load-displacement curves obtained from a three-point bend test for the IG300, NX9014, and OPC samples.

Specific Flexural Strength		
Class of materials	Symbols	Reference
Ceramics	▽	Precision Ceramics USA[57]
	□	Giordano *et al.*[32]
	○	Ruales-Carrera *et al.*[33]
	∗	Shah *et al.*[31]
Nacre	▽	Tremblay *et al.*[38]
	□	Loh *et al.*[30]
	○	Henry *et al.*[64]
	∗	Zhao *et al.*[1]
Cement/Concrete	▽	Liang *et al.*[46]
	□	Ghazanlou *et al.*[43]
	○	Liu *et al.*[47]
	∗	Subash *et al.*[48]
	+	Benazzouk *et al.*[41]
	◇	Chen *et al.*[39]
	☆	Zhang *et al.*[49]
	▷	Nam *et al.*[52]
	✡	Iqbal *et al.*[45]
PU Cement Composites	▽	Li *et al.*[51]
	□	Ding *et al.*[59]
	○	Hussain *et al.*[60]
	∗	Gao *et al.*[61]

Table S4: Class of materials, symbols and references used for specific flexural strength data represented in **Figure 7A**.

Thermal Conductivity		
Class of materials	Symbols	Reference
Ceramics	▽	Precision Ceramics USA [57]
	□	Cornacchia *et al.*[34]
	○	Wang *et al.*[35]
Nacre	▽	Tremblay *et al.*[38]
	□	Huang *et al.*[37]
	○	Li *et al.*[36]
Cement/Concrete	▽	Ghazanlou *et al.*[43]
	□	Liu *et al.*[47]
	○	Benazzouk *et al.*[41]
	∗	Chen *et al.*[39]
	+	Kurpinska *et al.*[42]
	◇	Janjoroen *et al.*[58]
PU Cement Composites	▽	Lanjekar *et al.*[56]
	□	Mounanga *et al.*[55]
	○	Gutiérrez-González *et al.*[65]

Table S5: Class of materials, symbols and references used for specific flexural strength data represented in **Figure 7B**.
